# Randomized controlled trial of a lay-facilitated angina management programme

**DOI:** 10.1111/j.1365-2648.2011.05920.x

**Published:** 2012-10

**Authors:** Gill Furze, Helen Cox, Veronica Morton, Ling-Hsiang Chuang, Robert JP Lewin, Pauline Nelson, Richard Carty, Heather Norris, Nicky Patel, Peter Elton

**Affiliations:** Coventry UniversityUK; York Trials Unit, Department of Health Sciences, University of YorkUK; Department of Health Sciences, University of YorkUK; Department of Health Sciences, University of YorkUK; British Heart Foundation Care and Education Research Unit, University of YorkUK; School of Community Based Medicine, University of ManchesterUK; Pennine Acute NHS TrustUK; NHS BuryUK; NHS BuryUK; NHS BuryUK

**Keywords:** angina nurses, cardiac rehabilitation, lay-led care, randomized controlled trial, self-management, stable angina

## Abstract

**Aims:**

This article reports a randomized controlled trial of lay-facilitated angina management (registered trial acronym: LAMP).

**Background:**

Previously, a nurse-facilitated angina programme was shown to reduce angina while increasing physical activity, however most people with angina do not receive a cardiac rehabilitation or self-management programme. Lay people are increasingly being trained to facilitate self-management programmes.

**Design:**

A randomized controlled trial comparing a lay-facilitated angina management programme with routine care from an angina nurse specialist.

**Methods:**

Participants with new stable angina were randomized to the angina management programme (intervention: 70 participants) or advice from an angina nurse specialist (control: 72 participants). Primary outcome was angina frequency at 6 months; secondary outcomes at 3 and 6 months included: risk factors, physical functioning, anxiety, depression, angina misconceptions and cost utility. Follow-up was complete in March 2009. Analysis was by intention-to-treat; blind to group allocation.

**Results:**

There was no important difference in angina frequency at 6 months. Secondary outcomes, assessed by either linear or logistic regression models, demonstrated important differences favouring the intervention group, at 3 months for: Anxiety, angina misconceptions and for exercise report; and at 6 months for: Anxiety; Depression; and angina misconceptions. The intervention was considered cost-effective.

**Conclusion:**

The angina management programme produced some superior benefits when compared to advice from a specialist nurse.

## Introduction

Stable angina is defined as pain or discomfort in the chest, jaw or shoulder that is evoked by exercise or emotion and is relieved by rest or nitroglycerin (Fox *et al.* 2006). It is considered stable when there is no increase in frequency or severity of symptoms (NICE 2011). Although it can be precipitated by a number of conditions, it is accepted that stable angina is a symptom of coronary heart disease (CHD). It is a chronic condition that affects over 2 million people in the UK (Scarborough *et al.* 2010), over six million in the USA (Roger *et al.* 2010), and has profound effects on functioning and quality of life (Lyons *et al.* 1994, Brorsson *et al.* 2002, MacDermott 2002, Spertus *et al.* 2002). Current guidelines for the treatment of stable CHD (including angina) emphasize the importance of encouraging people with heart disease to undertake secondary prevention programmes and to improve self management of their condition (Balady *et al.* 2007, NICE 2011). Cardiac rehabilitation is a comprehensive programme aimed at improving secondary prevention, physical and psychological functioning and quality of life, and which has been found in meta-analyses to reduce cardiac mortality after myocardial infarction (MI) by approximately 26%. Home-based versions of cardiac rehabilitation are as effective as centre-based for people post myocardial infarction or revascularization (Jolly *et al.* 2009, Dalal *et al.* 2010), and offering a choice of format can increase uptake among people with heart disease (Dalal *et al.* 2007). However, cardiac rehabilitation is often not routinely offered to people with stable angina; in the 2009 UK national audit of cardiac rehabilitation, 20% of all programmes actively excluded stable angina, and angina referrals accounted for only 4% of the 90,000 patients included in the audit (Lewin *et al.* 2010). Referral to and uptake of cardiac rehabilitation is also low in Europe, the USA, Canada and Australia (Cortes & Arthur 2006, Wenger 2008, Bakhai *et al.* 2011) with few data about stable angina, as it is not usually included in these audits.

## Background

Angina is a distressing condition; although its impact varies from person to person, over 50% of people with angina are limited in their activities, which can lead to premature retirement (Fox *et al.* 2006). A meta-analysis of seven trials of psychoeducational interventions for people with stable angina reported that such programmes reduced symptoms, improved quality of life and physical limitations (McGillion *et al.* 2008a). It has also been suggested that a number of common misconceptions about angina are associated with reduced physical and psychological functioning and quality of life in people with angina (Furze *et al.* 2003, 2005). Guidelines recommend that misconceptions about living with angina are dispelled [Scottish Intercollegiate Guideline Network 2007, NICE 2011]. According to a recent systematic review, programmes based on cognitive-behavioural principles may be effective in reducing these misconceptions (Goulding *et al.* 2010).

In a previous randomized controlled trial (RCT), it was demonstrated that a cognitive-behavioural, nurse-facilitated, angina self-management and rehabilitation programme (the Angina Plan) was significantly better than routine nurse education in reducing angina report and improving physical and psychological outcomes at 6-month follow-up (Lewin *et al.* 2002). A more recent study of the Angina Plan among people hospitalized with angina reported similar findings (Zetta *et al.* 2011). Since the publication of the results, over 900 professionals, the majority nurses, have been trained to deliver the Angina Plan, and over 20,000 patients have received the programme. However, as there are approximately 28,000 new cases of angina diagnosed in the UK each year (Scarborough *et al.* 2010), there are still many patients with stable angina who do not receive self-management education or rehabilitation, often due to lack of resources. The Angina Plan meets the UK standards (British Association for Cardiac Rehabilitation 2007) to be considered a form of home-based cardiac rehabilitation. The recent NICE guideline for the management of stable angina stated that ‘components of the Angina Plan were beneficial to people with stable angina but the evidence was not adequate to recommend the programme based on a small study sample’ (NICE 2011, p. 375).

Over the past decade there has been increasing report of self-management programmes delivered by peers or lay workers for people with long-term conditions (for example the studies by Lorig *et al.* 1994, Lorig *et al.* 1999, 2001a, Buszewicz *et al.* 2006, Kennedy *et al.* 2007). Indeed, the UK Department of Health (DH) highlighted self care as a ‘key building block’ in the National Health Service (NHS) Plan (DH 2000), and in ‘Self care; a real choice’ the DH further claimed that there was growing evidence of many benefits of supporting self care, including improvements in health and quality of life, and reduced use of health service resources (DH 2005).

In response to the report of lack of resources to deliver angina management or rehabilitation among primary care and community nurses that we talked to, and to the growing interest at that time in lay-facilitation of self-management support, we opted to develop a training programme for lay workers to become Angina Plan facilitators. As a consequence of the above-mentioned reported lack of resources, usual care following diagnosis in UK chest pain clinics is often simply one-off advice from an angina nurse specialist. We wanted to test whether or not lay-facilitation was more effective than usual care, as this would add to the evidence base for self-management programmes for people with angina.

## The study

### Aim

The aim of the study was to establish the relative effectiveness and comparative costs associated with a home-based, Lay-facilitated, Angina Management Programme (LAMP) when compared to routine advice and education from a specialist nurse.

### Design

**Figure 1:**
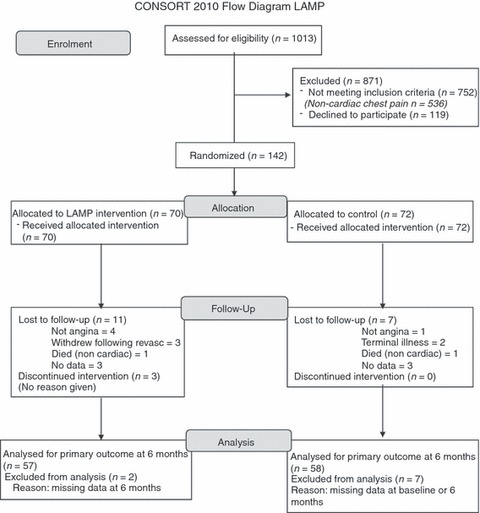
CONSORT diagram showing participant flow through the study.

This was a pragmatic RCT with allocation to either the LAMP or routine information and advice from a specialist angina nurse. The RCT was registered with the International Standard Randomised Controlled Trial Register: ISRCTN03137160, registered trial acronym – LAMP. All participants received care as usual from their GP and/or cardiologist. Figure 1 is a CONSORT diagram (Consolidated Standards of Reporting Trials) (Schulz *et al.* 2010) which shows the flow through the study.

#### Randomization and blinding

Remote telephone randomization using computer generated allocation was undertaken by people not involved in the study. Study measures were obtained by either postal questionnaire or by the research nurse who was not involved in the treatment. Baseline measures were obtained before randomization. Members of staff who delivered the treatments were not involved in data collection. Data entry was by automated scanner and all analyses were carried out blind to the randomization status.

### Participants

Recruitment was from all patients, diagnosed with angina in rapid access chest pain clinic (RACPC) at a district general hospital in the north of England, who met the inclusion criteria.

#### Inclusion

Adults (aged 18+ years) with a diagnosis of angina following a positive symptom-limited exercise treadmill test in RACPC; does not have any exclusion criteria.

#### Exclusion

Need for urgent revascularization, exercise induced arrhythmias or loss of systolic BP greater than 20 mmHg during exercise stress testing, self-report of rapidly increasing number and duration of attacks of angina; a score of 4 on the Canadian Angina Class or the New York Heart Association classification of heart failure; life-threatening co-morbidities; documented psychiatric problems (other than mild to moderate uni-polar depression or a simple anxiety state); dementia or confusion.

### Sample size

In the original study of the Angina Plan (Lewin *et al.* 2002) the mean change (sd) in angina frequency per week at 6-month follow-up for patients on the Angina Plan arm was –2·98 (5·54), and for the control patients was −0·40 (5·97). This gives an effect size of 0·45. To detect a standardized difference of 0·45 between the treatment groups in frequency of angina, with 80% power and 5% two-sided significance, required 158 participants to be included in the analysis.

### Details of the intervention and control arms

#### Intervention arm

**Figure 2:**
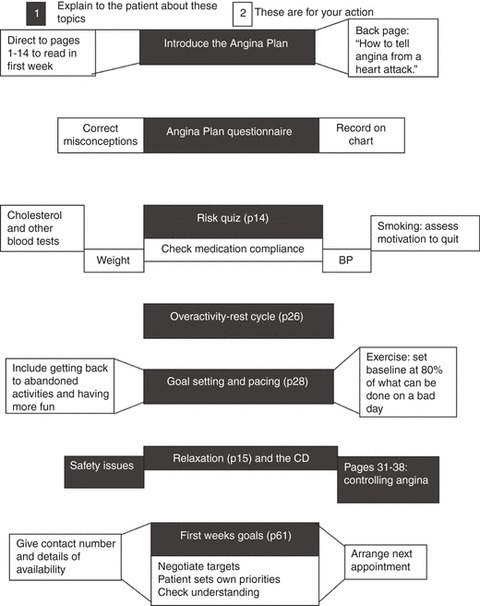
Angina Plan facilitation first interview flow chart.

The Angina Plan targets misconceptions about angina, and uses goal setting and pacing to increase activity and reduce risk factors. It includes a workbook and a relaxation programme on CD, and was introduced in a 45-minute interview by a lay facilitator during which misconceptions about living with angina were dispelled, and goals to increase physical activity and reduce behavioural risks for further heart disease were introduced. Stress management and other psychological issues were also raised (Figure 2 is a graphic representation of the flow through the first interview). Participants were shown how to record their progress, which was reported at follow-up in brief (10–15 minutes) telephone or home-visits, the number of which was negotiated between the facilitator and the patient. People who smoked were offered referral to the local Smoking Cessation Service. All lay facilitator follow-up was completed by 3 months after the initial interview.

The facilitators were six lay people with experience of heart disease, either personally (myocardial infarction and revascularization) or as carers of people with heart disease. They were recruited via advert in the local press, and were employed by the local NHS Primary Care Trust. The lay workers were four women and two men who were trained face-to-face, in 40 hours over 4 weeks with additional homework. They were managed by a community cardiac rehabilitation nurse; had regular group and individual supervision and were able to contact the nurse for advice at any time.

#### Control arm

Control group participants attended the clinic of the Angina Nurse Specialist, where the diagnosis was discussed and risk factor advice given, with referral to agencies such as the Smoking Cessation Service where appropriate. Participants also received written information about their condition and risk factors from sources such as the British Heart Foundation.

### Measures and outcomes

#### Primary outcome

Frequency of angina (assessed by a 1-week angina diary at baseline and 6 months).

#### Secondary outcomes

- Angina-related health status (including physical limitations, anginal frequency and perception and treatment satisfaction). This was measured by scales of the Seattle Angina Questionnaire (SAQ) UK – an angina-specific measure of health status that has been validated in a UK population (Garratt *et al.* 2001),
- Anxiety and depression measured by the Hospital Anxiety and Depression Scales (Zigmond & Snaith 1983).
- Angina-specific misconceptions measured using the York Angina Beliefs Questionnaire (YABQ) – a measure of common angina misconceptions that have been found to predict poor outcome (Furze *et al.* 2005),
- Risk factors (smoking status assessed by self-report and Bedfont carbon monoxide breath monitor, serum cholesterol, BP, BMI) and self-rated activity level [derived from the minimum dataset of the UK National Audit for Cardiac Rehabilitation (NACR) (Lewin *et al.* 2004)].
- Cost utility. This included the EQ-5D scores (Brook 1996) and measures of healthcare utilization (both elective and emergency), including GP visits, specialist nurse treatment, invasive tests and treatments (including angiography and revascularization), and their cost estimates were based on published sources [Personal Social Service Research Unit (PSSRU) 2009, DH 2008/9]. EQ-5D is one of the most widely used health related quality of life measures and gives summary index (utility) scores of health states for use in economic evaluation. The EQ-5D description system contains five dimensions – mobility, self care, usual activities, pain/discomfort and anxiety/depression; each dimension has three levels – no, some and severe problems. The costs of the intervention, including training and payment for lay workers, were estimated from trial records.

A cost for each trial participant was calculated as the product of resources used by their relevant costs over the period of 6 months. Two components were considered in the estimation of cost per participant – the cost of treatment and the cost of angina and heart related healthcare utilization. Apart from the resource use mentioned below, other resource uses were assumed to be equal across both treatment arms and thus not included in the following economics analyses.

### Validity and reliability

The NACR minimum dataset aims to give a standardized audit tool covering clinical, behavioural and health-related aspects of rehabilitation for UK cardiac rehabilitation programmes. The measures included in the dataset (including the HADS) were adopted following screening of over 200 measures for previous evidence of validity and reliability in UK cardiac populations, and assessment for acceptability among staff and patients in UK cardiac rehabilitation programmes (Lewin *et al.* 2004). The Seattle Angina Questionnaire scales and the angina diary were found to be reliable in the original Angina Plan study (Lewin *et al.* 2002). The YABQ was developed among angina populations in the UK and reported good internal consistency (Cronbach’s alpha 0·79–0·82) and stability (test–retest Pearson’s *r* = 0·79) (Furze *et al.* 2003).

### Recruitment procedure and data collection

Participants were recruited between May 2006 – September 2008, with follow-up complete in April 2009. The participants completed a 1-week angina diary after which the research nurse phoned the remote randomization service and arranged for a follow-up appointment with either the Angina Nurse Specialist (control arm) in outpatients’ clinic, or with a lay facilitator (intervention arm) to visit the participant at home, appropriately.

All data were collected at baseline and 6-month follow-up, questionnaire measures were also collected at 3 months. The 3-month follow-up was by postal questionnaires, and 6-month follow-up of all outcomes was undertaken by postal questionnaire and by a home visit from the research nurse to collect other outcome data.

### Ethical considerations

Ethical approval was granted prior to study commencement by an NHS Local Research Ethics Committee (05/Q1406/66). Approval for the study was also granted by the site Research Governance team.

### Data analysis

Analysis was conducted using the both Statistical Package for the Social Sciences (spss) version 16 and stata version 10 (StataCorp 2007, College Station, TX, USA). All analyses were by intention to treat. As the primary outcome total number of angina episodes at month 6 was count data and there was over-dispersion in the data, a negative binomial regression model was used to compare the two treatment groups. Intervention group was included in the model and the analysis was also adjusted for the number of angina episodes at baseline. The results are presented as incidence rate ratios.

The secondary outcome measures include; blood pressure, cholesterol, BMI, waist/hip ratio, SAQ and HADs. These were analysed by linear regression, the dependent variable being the outcome at month 3 or month 6. All analyses were adjusted for the baseline score of the dependent variable, including a term for the intervention group and controlling for the appropriate baseline value. Other secondary outcomes include smoking status at month 6 and whether or not the participant was undertaking the recommended level of exercise (5 × 30 minutes per week). These were both binary variables and so were analysed using a logistic regression model with the dependent data variable being the outcome at month 3 or month 6, the analyses were also adjusted for the baseline category, also included was a term for treatment group. Model checking was performed to ensure that the models were an adequate fit for the data.

### Cost-effectiveness analysis

A cost-utility analysis was conducted using stata 10. The health benefit of treatment was measured in units of quality adjusted life year (QALY). Utility was measured by the EQ-5D questionnaire at baseline and each follow-up of 3 and 6 months. The utility is derived from EQ-5D index score to which a pre-defined weight was assigned accordingly. The pre-defined weight represents the social preference of the general population in England and Wales towards EQ-5D health states. These utilities scores were then used to calculate QALYs. The incremental costs were compared with incremental QALY on an intention-to-treat basis. The adopted perspective was that of the UK NHS and Personal Social Service. The year of pricing was 2008.

The cost-effectiveness analysis used a net benefit regression approach with imputation of missing values, controlling for a number of covariates (age, gender, marital status, Canadian angina class, Charlson comorbidity score, smoker or not, over-weight or not, high cholesterol or not and EQ-5D index score). A willingness to pay of £20,000 per additional QALY was chosen (NICE 2008); if the incremental net benefit was greater than 0 the LAMP would be considered effective. To assess the level of uncertainty associated with the decision as to which intervention was most cost-effective, the cost-effectiveness acceptability curve (CEAC) is plotted (Van Hout *et al.* 2006). The CEAC shows the probability that the lay-facilitated angina management is more cost-effective than usual care, for different thresholds of willingness to pay for additional benefit (QALY).

## Results

Despite extending recruitment by 6 months, only 261 patients were eligible for the study, of whom 142 (54%) agreed to participate (70 randomized to LAMP and 72 to control). Baseline characteristics of the participants are shown in Table 1. At 6-month follow-up, data were collected from 124 (87%) of the participants, those continuing in the study were significantly younger than those who withdrew (62·9 vs. 71·5, 95% CI = 3·39–13·95) but there was no difference in gender or in number of angina episodes recorded at baseline.

**Table 1:** Baseline characteristics

	LAMP (*n* = 70)	Control (*n* = 72)
Age, Mean years (sd)	65·30 (9·66)	63·55 (10·19)
Female, *n* (%)	29 (41·43)	38 (52·78)
Marital status, *n* (%)		
Divorced	5 (7·25)	8 (11·11)
Married or in partnership	56 (81·16)	53 (73·61)
Single	2 (2·90)	1 (1·39)
Widowed	6 (8·70)	10 (13·89)
Canadian angina class, *n* (%)		
I	16 (22·86)	11 (15·28)
II	42 (60·00)	44 (61·11)
III	12 (17·14)	17 (23·61)
Work activity, *n* (%)		
Employed	21 (30·9)	17 (25·8)
Retired	36 (52·9)	41 (62·1)
Other	11 (16·2)	8 (12·1)
Charlson comorbidity score, Mean (sd)	0·37 (0·68)	0·35 (0·59)
Smoker current, *n* (%)	16 (22·86)	6 (8·33)
Systolic blood pressure, Mean (sd)	141·90 (19·01)	142·71 (16·48)
Total cholesterol, Mean (sd)	4·65 (1·11)	5·33 (1·24)
Body mass index, Mean (sd)	28·73 (6·34)	27·97 (4·64)
Waist/hip ratio, Mean (sd)	0·98 (0·13)	0·95 (0·10)
Angina episodes, count over 1 week, median (25–75 percentile)	3 (0–5)	2 (0–8)

The primary analysis did not find any important differences in the rate of angina between the treatment groups at month six. The rate was measured over a sample week. The incidence rate ratio for Control (*n* = 57) vs. LAMP (*n* = 58) was 0·96 (95% CI: 0·39–2·38; *P* = 0·926), compared to LAMP, the control group had an angina rate over the sample week of 0·96 times less than the LAMP group. This difference was not statistically significant.

At 6 months, the median number of angina episodes over 1 week was nil in both groups, a reduction from a median of 3 at baseline for LAMP group and 2 for control group. The proportion of patients that were angina free at 6 months was 75% for the LAMP group and 62% for the control group.

Of the secondary outcomes (Table 2), there were important differences in favour of the LAMP group for: waist-to-hip ratio at 6 months, anxiety at 3 and 6 months, depression at 6 months but not at 3 months, and angina misconceptions at both time points. Significantly more of this group also reported meeting guideline amounts of exercise at 3 months but not at 6 months. The LAMP group had significantly higher quality of life as measured by EQ-5D index scores, at both 3 months [mean (sd) = 0·82 (0·21) vs. 0·70 (0·28) *P* = 0·01] and at 6 months [mean (sd) = 0·82 (0·24) vs. 0·68 (0·32) *P =* 0·008]. The remaining outcomes were not significantly different between the two groups.

**Table 2:** Secondary outcomes

	LAMP (*n* = 57), Mean (se)	Control (*n* = 58), Mean (se)	Difference, Mean (95% CI)	*P*
Physiological measures at 6 months				
Systolic blood pressure*	136·99 (2·30)	137·95 (2·22)	−0·96 (−7·30, 5·37)	0·76
Total cholesterol*	4·27 (0·14)	4·15 (0·13)	0·11 (−0·28, 0·50)	0·57
Body mass index*	28·05 (0·68)	29·03 (0·66)	−0·98 (−2·86, 0·89)	0·30
Waist/hip ratio*	0·95 (0·01)	0·98 (0·01)	−0·03 (−0·05. 0·00)	0·05
Questionnaire measures at 3 and 6 months				
SAQ Physical limitations 3 months*	83·08 (2·28)	78·89 (2·25)	4·19 (−2·16, 10·55)	0·19
SAQ physical limitations 6 months*	81·30 (2·32)	81·23 (2·14)	0·07 (−6·18, 6·33)	0·98
SAQ anginal frequency/perception 3 months*	71·52 (3·16)	63·20 (3·13)	8·31 (−0·52, 17·14)	0·07
SAQ anginal frequency/perception 6 months*	74·70 (3·24)	70·62 (3·06)	4·08 (−4·76, 12·93)	0·36
SAQ treatment satisfaction 3 months*	85·95 (2·86)	82·13 (2·86)	3·81 (−4·19, 11·82)	0·35
SAQ treatment satisfaction 6 months*	89·48 (2·42)	86·13 (2·24)	3·35 (−3·20, 9·89)	0·31
HADS anxiety 3 months*	5·13 (0·39)	7·07 (0·38)	−1·94 (−3·03, −0·84)	0·001
HADs anxiety 6 months*	6·27 (0·46)	7·70 (0·44)	−1·43 (−2·69, −0·17)	0·03
HADS depression 3 months*	3·40 (0·28)	4·05 (0·28)	−0·65 (−1·45, 0·14)	0·11
HADs depression 6 months*	3·11 (0·39)	4·21 (0·38)	−1·10 (−2·19, −0·02)	0·05
Angina beliefs 3 months*	4·28 (0·26)	5·15 (0·25)	−0·86 (−1·58, −0·15)	0·02
Angina beliefs 6 months*	4·17 (0·22)	5·63 (0·22)	−1·47 (−2·09, −0·85)	<0·001

Categorical variables smoking (verified by CO Monitor)	LAMP, *n* (%)	Control, *n* (%)	Odds ratio (95% CI)	*P*
6 months†	12 (22·2)	6 (10·3)	1·31 (0·11, 15·32)	0·83
Exercise 5 × 30 minutes per week				
3 months†	30 (53·6)	18 (32·1)	3·14 (1·30, 7·58)	0·01
6 months†	22 (38·6)	23 (36·5)	1·12 (0·50, 2·49)	0·78

All results adjusted for baseline value of the dependent variable.

SAQ = Seattle Angina Questionnaire, higher scores = better functioning; HADS = Hospital Anxiety and Depression Scales, higher scores = worse functioning; Angina beliefs, higher scores = more misconceptions.

* Estimates obtained from linear regression model.

† Estimates obtained from logistic regression model.

### Resource use and cost

#### Cost of treatment – intervention

A total of six lay facilitators were recruited in the LAMP trial and each cost £179 for training. The cost of a lay worker per hour was £6·15 whilst the travel costs per home visit was assumed at £5 per trip. The cost of per telephone call (for 15 minutes) and a supporting material (workbook) were £0·05 and £10, respectively. The frequency and durations of home-visits and phone calls per patient received were recorded during the follow-up period by their lay workers.

#### Control

A 20 minutes meeting with a cardiac rehabilitation nurse specialist cost £12·67. Table 3 presents healthcare utilization by each follow-up and group. There were no important differences between the groups for GP visits or hospitalizations at 3 or 6 months. In terms of total cost calculation (imputed and adjusted), the cost of healthcare utilization per patient was summed by the cost of intervention or the cost of control treatment. In this regression model, the average cost per patient in the control group was £1259·9 (CI: 764·5–1755·4) whilst in the intervention group it was £1496·0 (CI: 901·0–2091·0). The difference between the two groups was not significantly different from zero (£236·0, CI: −825·5–1001·6).

**Table 3:** Healthcare utilization in each follow-up and group

	LAMP (*n* = 70)	Control (*n* = 72)
*3-month follow-up*		
GP visit		
Mean (sd)	0·6 (1·7)	0·4 (1·1)
Median (Min–Max)	0 (0 – 10)	0 (0 – 4)
Missing (%)	14 (22·9)	14 (19·4)
Hospitalization, the number of cases		
Chest pain	2	1
Angiogram	4	8
PCI	2	6
CABG	1	0
Missing (%)	14 (22·9)	14 (19·4)
*6-month follow-up*		
GP visit		
Mean (sd)	0·4 (1·0)	0·2 (0·7)
Median (Min–Max)	0 (0–6)	0 (0–3)
Missing (%)	9 (15·7)	7 (9·7)
Hospitalization, the number of cases		
Chest pain	5	1
Angiogram	0	1
PCI	4	3
CABG	6	5
Missing (%)	9 (15·7)	7 (9·7)

PCI, percutaneous coronary intervention; CABG, coronary artery bypass graft surgery.

### Assessment of cost utility

There was a statistically significant difference in average QALY per patient of 0·045 (CI: 0·005–0·085). It was estimated from the model that, after adjusting for covariates, the average incremental net benefit of LAMP over control was positive (£354·60) and therefore LAMP can be considered cost-effective. However, the coefficient was not significantly different from zero (*P* = 0·408), representing some level of uncertainty around this net benefit estimate.

**Figure 3:**
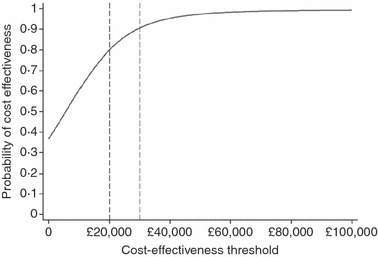
Cost-effectiveness acceptability curve.

The cost-effectiveness acceptability curve (Figure 3) shows the probabilities representing the chance that LAMP is more cost-effective compared to control for a range of willingness to pay (λ). As seen in figure 3, the probability increased with the increasing value of λ. For λ = £20,000 the probability of LAMP being cost-effective is 80%. For λ = £30,000 the probability increased to 90%.

## Discussion

### Limitations of the study

The major limitation of the study is that it did not recruit enough participants to meet the sample size requirements, and is therefore underpowered to show differences. The main reason for the lack of recruitment was the unexpectedly large number of people who did not meet inclusion criteria (871 of 1013 people attending clinic, the majority of whom were recorded as having non-cardiac chest pain). Whilst we were expecting that 50% would not be eligible, based on previous audit within the same RACPC, the additional numbers of ineligible patients reduced the pool of prospective participants who could be recruited within the time frame. In addition, we have no details of the people who refused to participate, and so we cannot be certain that the study population fully reflects data of all people diagnosed with stable angina in RACPC.

The study did recruit the same number of participants as in the original study, and does demonstrate some important differences between the two groups. This was a pragmatic randomized trial, conducted in the appropriate clinical settings, thus increasing the likelihood that its findings would apply to real practice. The quality of the study was high; randomization was by remote centre, data were collected by a nurse not involved in delivering the interventions and analysis was blind to group; however, participants could not be blinded their randomization status. A further strength is the high retention rate – with 87% completing follow-up at 6 months. In addition, the costs of both interventions have been estimated and analysed.

### Discussion of the results

There was no difference in angina frequency between the groups. This difference is likely to be due to improvements in modern drug therapy, as the majority of participants did not have any angina at follow-up, which contrasts not only with the report of angina at follow-up in the original study (Lewin *et al.* 2002) but also with the studies reported in the meta-analysis of psychoeducational interventions.(McGillion *et al.* 2008a). However, as shown in Table 3, this lack of difference in angina frequency was not due to revascularization with percutaneous coronary intervention (*N* = 15) or coronary artery bypass graft surgery (*N* = 12), as relatively few participants received these interventions during the time frame. The lack of angina symptoms would also explain the lack of difference between the two groups in the Seattle Angina Questionnaire scales, which report the effect of angina on physical and psychological functioning. The LAMP had little extra effect on risk factors compared to advice from a nurse specialist; the exceptions being self-reported exercise at 3 months (not maintained to 6 months), and 6 month waist-to-hip ratio. The LAMP did have important effects on anxiety and misconceptions at both follow-up time points, and on depression at 6 months. LAMP is cost-effective.

There are very few studies of angina self-management programmes, and therefore comparison with the literature is limited. In the original Angina Plan study (Lewin *et al.* 2002) there was an important effect on angina frequency which was not matched by lay-facilitation in this study. This difference is likely to be due to improvements in modern drug therapy, as the majority of patients were angina-free at 6-month follow-up. However, it is also possible that the populations differed between the two studies. For this study, patients were recruited following diagnosis of stable angina within RACPC. This means that their symptom onset was often <1 month. As there were very few RACPCs in 2000, the original study recruited people from primary care, where the most efficient method of identification (given known problems with diagnostic codes) was prescription of short-acting nitrates. All patients had their symptoms for <1 year, but their use of nitrates suggests that they were still suffering episodes of chest pain, and so may have been more refractory than the participants in the current study.

**What is already known about this topic**

- Guidelines recommend that people with stable angina are supported to improve their condition self-management and secondary prevention of heart disease, but only a minority are referred to such programmes.
- Increasingly, self-management support provided by lay workers is employed among people with long-term conditions.
- A randomized controlled trial of a nurse-facilitated angina management programme reported important improvements in report of angina and quality of life when compared to routine nurse advice and education.

**What this paper adds**

- Lay workers can be trained to facilitate a lay angina management programme, under the supervision of community cardiac rehabilitation nurses.
- Compared to a single advice and education interview from an angina nurse specialist, a 12-week lay-facilitated angina management programme had important effects on reports of exercise and psychological functioning, but no effect on report of angina and is considered cost-effective.
- This study gives further evidence that lay-delivered self-management support in long-term conditions produces modest benefits.

**Implications for practice and/or policy**

- Where a full nurse-led angina management programme is not possible due to resource issues, it may be possible that a combined nurse/lay service will produce the necessary clinical improvements in secondary prevention, while providing the psychological support shown in this study.

The effects on secondary outcomes in this study were also not as profound as those in the original study. There was an increase in activity among the LAMP group at 3 months (when lay facilitator support ceased) which was not maintained to 6 months, whereas improvements in physical functioning were found at 6 months in both previous studies (Lewin *et al.* 2002, Zetta *et al.* 2011). It may be that facilitation of the Angina Plan by a nurse produces longer lasting change in health behaviour for exercise than facilitation by a lay person. McGillion *et al.* (2008c) developed a nurse-facilitated, group-based angina self-management programme derived from the Chronic Disease Self-management Programme developed by Kate Lorig at Stanford University (Lorig *et al.* 2001b) and tested this in a RCT with 130 participants. The Chronic Angina Self-Management Programme (CASMP) was compared to usual care (waiting list) and followed up for 3 months, It was found to reduce angina frequency and improve physical functioning but had no effect on psychological functioning as measured by the Short-Form 36 questionnaire (McGillion *et al.* 2008c). The combined evidence from the three studies of nurse-facilitated self-management programmes (Lewin *et al.* 2002, McGillion *et al.* 2008c, Zetta *et al.* 2011) may suggest greater effect on outcome of nurse delivered care, however, this would need to be tested in a randomized trial. As lay Health Trainers are increasingly delivering health behaviour change advice to people with long-term conditions in the UK, the discrepancy in the uptake of exercise following lay or nurse facilitation requires further investigation in studies directly comparing the two methods of delivery of self-management programmes. In addition, follow-up needs to be longer than 3 or 6 months, as requested by NICE (2011).

With the exception of waist-to-hip ratio, there were no other important effects on risk factors in this study, whereas Zetta *et al.* study reported a difference in BMI (Zetta *et al.* 2011). However, this difference in outcomes may reflect the lack of power of the present study, as there was a greater reduction in BMI among the LAMP participants than among those in the Angina Plan group in the Zetta *et al.* study.

Participants in the LAMP reported significantly less anxiety at both time points and less depression at 6 months, which compares with the findings of the original study, but not that of Zetta *et al.*(2011) (which found no effect on anxiety and depression). However the participants in the Zetta *et al.* study had very low levels of anxiety and depression at baseline (mean scores of <3 for both HADS Anxiety and HADS Depression) and therefore it would be difficult to achieve important reductions in these scores. The meta-analysis of psychoeducational programmes was unable to pool estimates of effects on psychological well-being due to heterogeneity (McGillion *et al.* 2008a).

The burden of angina on patients is large; McGillion *et al.* (2008b) estimated that the total annual cost of living with angina was $19,209. As the cost per QALY for people receiving LAMP is much lower than the widely accepted value of £20–30,000 per QALY, which seems to influence NICE decision-making, it would seem that LAMP is cost-effective. A similar nurse-facilitated programme for people awaiting coronary artery bypass graft surgery also was found to be cost-effective (Furze *et al.* 2009). Other studies of angina rehabilitation have produced no figures of cost utility, and therefore comparisons are not possible. The study by McGillion *et al.* did not assess cost utility of the CASMP, instead they assessed whether the programme reduced the financial burden of people with stable angina, but there was no effect.

People with stable angina need support to reduce their risk of further cardiac events, and this study adds to the evidence of nurse involvement in promoting self-management skills in their patients. It must be remembered that the lay workers in this study were managed by the community cardiac rehabilitation nursing team who provided supervision and clinical support. In addition, the evidence for nurse-facilitated or supported self-management programmes has been developed in two different health services (Canada and the UK), suggesting that such programmes can be incorporated internationally into the nursing care of people with stable angina, although further evidence is required to support this suggestion.

## Conclusion

This study showed that, although the LAMP produced some benefits when compared to advice from an Angina Nurse Specialist, particularly on anxiety and depression, the benefits were not as profound as those previously produced by nurses facilitating the same programme. People may feel greater motivation to change their risk behaviour if they report these changes to a health professional. However, the LAMP was cost-effective, and it may be that a service where the programme is introduced by a nurse but follow-up is by lay facilitators would produce a greater impact on risk factors while maintaining the psychological support that is evident here. Such a skill-mix may produce similar outcomes to a fully nurse-facilitated programme but at less cost – however this would need testing for confirmation of this hypothesis.

NICE has recently confirmed that there are very few studies of the efficacy and cost utility of cardiac rehabilitation or self-management in people with stable angina (NICE 2011), and therefore this study will add to that evidence base. In addition, there have been no trials of group vs. home-based cardiac rehabilitation in this patient group. This emphasizes the need for well-designed and fully powered, multi-centre studies of both self-management and of angina rehabilitation, a point endorsed in the NICE guideline (NICE 2011).
